# A Messianic Trinity

**Published:** 1903-11

**Authors:** 


### A MESSIANIC TRINITY.

THE fizzle of the invasion of New York by Dowie and the Hosts of Zion shows a degree of scepticism towards reincarnation and the Messianic idea that should discourage other aspirants for the distinction. Dowie flapped about on the stage like a crippled buzzard, now and then spattering the audience with showers of filthy vituperation. If the real Elijah were fed by the ravens, it would seem from the character of the fake prophet's utterances that buzzards had assumed for him and his hosts commissary functions. He announces his intention of returning to the wilderness where his providers, the winged scavengers of the air, may wheel and soar foraging, without having their job filched from them by aromatic garbage scows. After the dramatic ascension of the first Elijah it would be a pity for Elijah II. to have to return to Zion in a cattle car. But the quicker he returns to his native fastnesses and proceeds to warm over his old converts without striving after new ones the better it will be for J. Aleck Dowie and his followers.

For there are other claimants for Messianic honors. Mr. Pigott, of Cedar Lodge, Clapton, London, goes Dowie several better—that is, when judged by the arrogance of his pretensions. He announces that he is the very Messiah himself, and more than that, that he is God. Of course he has followers. However vain and blasphemous his assertions there are always those whose credulity is equal to the task. The insane asylum yearns for such aberrants, for it always holds among its number a miniature Olympus.

Again, from far off India is heard the voice of one crying in the wilderness. "The promised Messiah" asserts that God raised him in the land of the Punjab, for his works are wonderful. "I am the true Messiah who has come in the last ages." The evidence of this assertion is manifested in hundreds of heavenly signs. The face of God is revealed in the "looking glass of his person." Further substantiation of the claim will appear in the death of Pigott, and Dowie shall leave the world in great sorrow and torment. Jesus did not die on the cross, but having escaped from it with his life, subsequently died at Srinagar (Kashmir) and his tomb may still be seen in the Khan Yar street. This last statement is a powerful argument in favor of the "Promised Messiah," for it is eminently natural and plausible that resurrection should occur as near as convenient to the place of interment, and the "Promised Messiah" was raised in the land of the Punjab.

People of the class of Dowie, Pigott and the Indian fakir are greedy and scheming impostors who thrive and grow rich on the credulity of the ignorant and emotional by operating through the mysteries of religiosity, or else they are self-deceived fanatics with delusions. In either instance, they soon exhaust themselves and perish. There are enough sane and temperate people in the world to carry on its serious affairs. They stand by and witness the tricks and antics of the newest claimant to such notoriety much with the same contemptuous amusement as when viewing a slap-stick comedy. It is rasping, however, when such callow absurdities invade the dignified and sanctified precincts of our faith.

THE Board of Governors of the Georgia Pasteur Institute held their annual meeting in Atlanta October 3. Dr. Henry R. Slack, the president, read his third annual report, which showed that they have treated 150 persons bitten by animals supposed to be rabid and have lost only one. This is a splendid showing, and is surpassed, we believe, by no other institute in this country. The following officers were elected; President, Dr. Henry R. Slack, LaGrange; Vice-President, Dr. J. H. McDuffie, Columbus; Treasurer, Dr. C. D. Hurt; Secretary and Physician, Dr. J. N. Brawner; Assistant Physician, Dr. John S. Hurt; Pathologist, Dr. Claude A. Smith.

---

RECENTLY in Atlanta a Miss Flynt married a Mr. Steele. The sparking, however, preceded the union, which was solemnized in due form by one of the big guns in the Methodist church and did not, like so many affairs of the heart, flash in the pan. The tender flame has ere this softened the heart of flint and bent the resisting steel.

---

WE publish elsewhere a letter from Dr. Lydston, of Chicago. We trust the doctor who is accused of piracy will be able to explain the affair and acquit himself of the charge, as no doubt he will be able to do.

---

THE medical department of Kentucky University has decided to introduce what is known as the quarter system, by which a student may attend the school the whole year or at any time of the year. Two terms of three months each make up a year's course, but a student may begin with any quarter, or in fact attend all four if he wish. The authorities believe this system will be taken up by other medical schools.—*New York Medical Journal.*

